# RAS, Cellular Plasticity, and Tumor Budding in Colorectal Cancer

**DOI:** 10.3389/fonc.2019.01255

**Published:** 2019-11-19

**Authors:** Valeria Maffeis, Lorenzo Nicolè, Rocco Cappellesso

**Affiliations:** ^1^Department of Medicine, Surgical Pathology and Cytopathology Unit, University of Padova, Padova, Italy; ^2^Pathological Anatomy Unit, Padova University Hospital, Padova, Italy

**Keywords:** RAS, colorectal cancer, plasticity, epithelial to mesenchymal transition, tumor budding

## Abstract

The high morbidity and mortality of colorectal cancer (CRC) remain a worldwide challenge, despite the advances in prevention, diagnosis, and treatment. RAS alterations have a central role in the pathogenesis of CRC universally recognized both in the canonical mutation-based classification and in the recent transcriptome-based classification. About 40% of CRCs are *KRAS* mutated, 5% *NRAS* mutated, and only rare cases are *HRAS* mutated. Morphological and molecular correlations demonstrated the involvement of RAS in cellular plasticity, which is related to invasive and migration properties of neoplastic cells. RAS signaling has been involved in the initiation of epithelial to mesenchymal transition (EMT) in CRC leading to tumor spreading. Tumor budding is the morphological surrogate of EMT and features cellular plasticity. Tumor budding is clinically relevant for CRC patients in three different contexts: (i) in pT1 CRC the presence of tumor buds is associated with nodal metastasis, (ii) in stage II CRC identifies the cases with a prognosis similar to metastatic disease, and (iii) intratumoral budding could be useful in patient selection for neoadjuvant therapy. This review is focused on the current knowledge on RAS in CRC and its link with cellular plasticity and related clinicopathological features.

## Introduction

Colorectal cancer (CRC) is a malignant epithelial tumor originating in the large bowel and in almost all cases it features as an adenocarcinoma, a neoplasia with glandular characteristics (1). Despite the big efforts of the last decades resulting in the widespread implementation of screening programs, that have proved effective in reducing the burden of the disease in the population, and in the advances of the surgical and systemic treatments, that have improved the outcome of the patients, CRC is still the third cancer for incidence and the second for mortality in both sexes worldwide (2–4). This highlights the urgent need to identify novel diagnostic, prognostic, and predictive markers and to develop new strategies for CRC prevention, early detection, and therapy to drastically reduce CRC morbidity and mortality. Indeed, the identification of circulating markers would allow to anticipate the identification of CRC in the population, to early detect interval cancers, and to better select patients really needing colonoscopy. The current categorization based on tumor histology, grade, and stage provides limited understanding of CRC biology and often fails to recognize the true high-risk population after surgery. Consistent prognostic markers would allow to tailor the treatment according to the aggressiveness of the tumor. The development of reliable sentinel lymph node methods would modify the surgical management of the disease. The discovery of mechanisms impairing the response to current drugs and of novel targetable molecular alterations would allow a more appropriate therapy in specific subgroups of patients.

In the past, pioneering morphological, and molecular studies allowed to disclose the chain of events underlying the “adenoma to carcinoma cascade” theorized by Fearon and Vogelstein characterized by chromosomal instability (CIN) and sequential mutations of Adenomatous Polyposis Coli (*APC*), Kirsten rat sarcoma viral oncogene homolog (*KRAS*), and tumor protein p53 (*TP53*) genes (5). The importance of this model is such that it is the foundations on which CRC secondary prevention is based. However, it was soon clear that this model of carcinogenetic progression was not applicable to all cases of CRC since it is a heterogeneous disorder with a great variability in response to the therapies and presumed to arise from distinct precursor lesions (6). Subsequent molecular studies led to the identification of various subtypes of CRC, then grouped into a mutation-centered classification (6). However, even this approach partially failed to grasp the biological behavior of CRC and was inadequate in explaining the diversity in patient outcomes (7). More recently, research focused on gene expression profiling and characterization of tumor microenvironment pressures and stimuli to try to fill the gap in the understanding of the disease. Such strategies deepened the knowledge about cellular mechanisms of tumor progression, allowed to discover novel morphological clues of cancer aggressiveness, and provided a huge amount of data finally condensed in a new molecular classification (8).

In this article, we summarize the most meaningful molecular classifications of CRC highlighting the role of RAS in this tumor and its link with cellular plasticity, invasion, and migration at both molecular and morphological levels.

## Molecular Classifications of Colorectal Cancer

In the “adenoma to carcinoma” model, CRC carcinogenesis is presented as a stepwise process based on the accumulation of molecular alteration contributing to the malignant transformation of the mucosa. In this cascade, *APC* inactivation initiates the evolution of the mucosa into the adenoma and subsequent *KRAS* and *TP53* mutations drive the emergence of increasingly aggressive subclones (5). However, the evidence that a consistent number of CRCs lacks *APC* and *KRAS* mutations has slowly eroded the foundations of this linear theory. Thus, a different categorization was needed because tumor classification is not just to give a name to the entities, but to differentiate them according to the clarification of the clinicopathological correlations, the determination of the etiologies, and the understanding of the evolution of the disease to achieve the best response to treatment.

**Figure 1:**
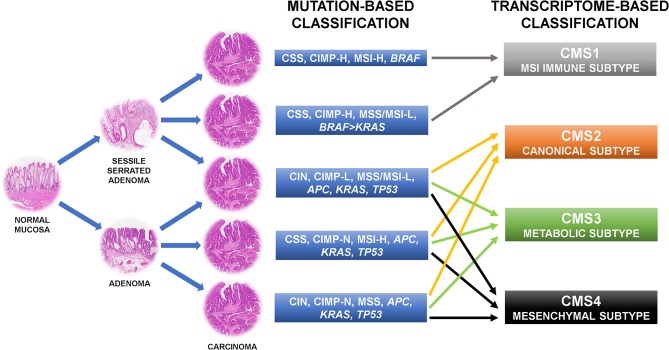
Colorectal cancer molecular classifications recently shifted from the mutation-based toward the transcriptome-based approach because this can better describe the behavior of the tumors. CIN, chromosomal instability; CSS, chromosomal stability; CIMP-N/L/H, CpG island methylator phenotype-negative/low/high; MSS, microsatellite stability; MSI-L/H, microsatellite instability-low/high.

The first attempt to organize CRC subgroups based on correlation of clinical, morphological, and molecular features used two main molecular alterations: genetic instability and DNA methylation (6, 9–11). Genetic instability can occur in two mutually exclusive forms, one affecting whole chromosomes or portions of chromosomes (namely CIN), the other affecting small repetitive sequences of DNA [namely DNA microsatellite instability (MSI)] (12). Thus, a CRC with CIN is DNA microsatellite stable (MSS). MSI was further stratified in MSI-high (MSI-H), and MSI-low (MSI-L) depending on the frequency of the mutations in the repetitive DNA sequences throughout the genome (13). These two conditions are also linked to different onset mechanisms. While MSI-H is related to the loss of expression of one or more members of the DNA mismatch repair machinery (namely MLH1, MSH2, MSH6, and PMS2), MSI-L is connected to extensive DNA methylation of the genome due to partial methylation and loss of expression of MLH1 or loss of expression of 0-6-Methylguanine DNA Methyltransferase (MGMT) (14–16). Epigenetic instability due to aberrant promoter CpG island hypermethylation is the second cornerstone on which CRC classification is based. According to the frequency of methylation of CpG loci, CRCs are separated into negative, low, and high CpG island methylator phenotype (CIMP) groups (17–20). The combination of these features results in a classification outlining five molecular subgroups of CRC whose alterations can be found also in definite precancerous lesions (Figure 1). The first subtype is the conventional CRC originating from adenoma. The tumor may be sporadic or associated with inherited conditions such as familial adenomatous polyposis (FAP) and *mutY DNA glycosylase* (*MUTYH*)-associated polyposis (MAP) (21). It is the most common type of CRC accounting for ~57% of cases and is molecularly characterized by CIN, CIMP negativity, and MSS. *APC, KRAS*, and *TP53* genes are usually mutated, accordingly to the “adenoma to carcinoma” sequence (6). Another CRC subtype following this mutational cascade is represented by tumors developing from adenomas in the context of Lynch syndrome (accounting for about 3% of CRCs). Indeed, these tumors are chromosomal stable and CIMP-negative, but have a hypermutator phenotype due to MSI-H caused by the inherited mutation affecting one or more components of the DNA mismatch repair system (22). *BRAF* gene is typically wild type, as opposed to the so-called sporadic MSI-H CRC that is characterized by chromosomal stability, CIMP-H, *MLH1* methylation, MSI-H, and *BRAF* mutation (23). This sort of CRC accounts for about 12% of cases and is thought to derive from sessile serrated adenoma (6, 23, 24). Another subgroup of CRC (about 8% of cases) originating from sessile serrated adenoma has chromosomal stability, CIMP-H, only partial methylation of *MLH1*, MSS or MSI-L, and harbors more commonly mutation of *BRAF* than of *KRAS* (6). The last subtype of CRC may develop from both conventional adenoma and sessile serrated adenoma, includes about 20% of tumors, and is characterized by CIN, CIMP-L, MSS, or MSI-L due to MGMT methylation, and always *KRAS* mutations (6). In general, CRCs with CIN are relatively more aggressive than those with MSI (25–27) and CIMP-H tumors has a less favorable prognosis than CIMP-L ones, but if CIMP-H is associated with MSI-H the outcome is slightly better (28, 29). Moreover, MSI CRCs are known to be not responsive to adjuvant fluorouracil-based therapy but may benefit of immune checkpoint blockade with anti-PD1 immunotherapy (30, 31). The major limit of this categorization is that tumors in each subgroup are considered to be a homogeneous entity from a therapeutic point of view, however they show profound differences in drug response and prognosis.

For this reason, more recent approaches shifted from the mutation-based toward the transcriptome-based classification thinking that it can better describe the behavior of the tumors. Indeed, several of such categorizations found CRC gene expression profiles more adherent to the outcome of the patients than the previous system (7, 32–37). These patient stratifications could be useful for the therapeutic decision-making process and are attractive for a rapid translation into the clinic, thus there are many expectations in this regard (7). However, several inconsistencies have emerged by the comparison of the results of these new classification systems. Indeed, each study has attained its own taxonomy including a different number of CRC subtypes. These substantial discrepancies were mostly due to the different CRC populations investigated, the various analysis platforms used, the distinct methods of bioinformatic analysis applied, and the interpretation of data performed (7, 32–37). To clear these hurdles, the CRC Subtyping Consortium (CRCSC) was formed with the purpose of evaluating potential overlaps among the different transcriptome-based CRC classifications to identify core subtype patterns (Figure 1) (8). Four consensus molecular subtypes (CMSs) were delineated using a network-based meta-analysis method of six different taxonomies followed by comprehensive multi-omic and clinical characterization (8). The CMS1 sort of CRCs accounts for about 14% of cases and corresponds to the “MSI immune subtype” characterized by MSI, CIMP-H, *BRAF* mutations, and intense and widespread immune infiltrate (8). CSM2, the so-called “canonical subtype,” is the most common subtype of CRC accounting for ~37% of tumors. Epithelial characteristics, CIN, activation of WNT and MYC signaling pathways, and upregulation of the miR-17-92 cluster feature this CRC (8). About 13% of CRCs are included in the “metabolic subtype” or CMS3 group, characterized by loss of regulation of metabolic pathways, CIN, CIMP-L, heterogeneous MSI-status, *KRAS* mutations, and let-7 miR family downregulation (8). Overexpression of epithelial to mesenchymal transition (EMT) markers, miR-200 family downregulation, activation of TGF-β pathway, neoangiogenesis, and stromal infiltration feature the CRC subgroup related to the worst prognosis: the “mesenchymal subtype,” namely CMS4 (8). This subtype accounts for about 23% of CRC cases. Of note, ~13% of CRCs are not classifiable in any of these categories because of intratumoral heterogeneity or a phenotype mixing molecular features of several CMS subtypes (8). The frequency of *KRAS* mutation varies among the CRC subtypes (23% in CMS1, 38% in CMS2, 28% in CMS3, and 68% in CMS4) and this could explain the different behavior of mutated tumors (7).

## RAS in Colorectal Cancer

The human *RAS* gene family includes three members, namely *KRAS*, neuroblastoma RAS viral oncogene homolog (*NRAS*), and Harvey rat sarcoma viral oncogene homolog (*HRAS*), encoding four proteins: KRAS4A and KRAS4B (secondary and prevalent isoforms, respectively, deriving from alternative splicing of the RNA), NRAS, and HRAS (38). By means of their GTPase enzymatic site, these small proteins play as molecular switches transducing extracellular signals, such as growth factors, differentiation factors, and mitogens, to transcription factors and cell cycle proteins in the nucleus thus triggering cell growth, differentiation, proliferation, and survival. This site cycles between the guanosine diphosphate (GDP)-bound inactive and the guanosine-5′-triphosphate (GTP)-bound active forms. In normal conditions, extracellular cues stimulate transmembrane tyrosine kinase receptors which recruit guanine nucleotide exchange factors (RASGEFs) promoting activation of the RAS GTPase through the hydrolysis of GDP to GTP (39). In turn, RAS recruits and activates several downstream effectors in different pathways, mainly the phosphoinositide 3-kinase (PI3K)-AKT pathway and the cascade comprising RAF kinase, which activate mitogen-activated protein kinase kinases 1 and 2 (MEK1 and MEK2), and subsequent activation of extracellular signal-regulated kinases 1 and 2 (ERK1 and ERK2), thus promoting cell survival, proliferation, invasion, and migration (39, 40). Missense gain-of-function mutations in members of the *RAS* family have been found in about 25% of all human cancers. Usually, these are single nucleotide point mutations involving few hotspot regions: the codons 12 and 13 in exon 2, the codons 59–61 in exon 3, and the codons 117 and 146 in exon 4. Such mutations result in a conformation of the RAS active site having intrinsic hydrolytic capability (39). Thus, in mutated cells occurs an accumulation of constitutively GTB-bound active RAS proteins able to trigger downstream signaling even in the absence of extracellular stimuli.

*KRAS* is the most frequently mutated isoform accounting for about 20% of all human cancers. NRAS and HRAS mutations, instead, are found in about 8 and 3% of cancers, respectively (39). Interestingly, different cancer types are related to mutation of a precise RAS isoform, suggesting that the carcinogenetic role of RAS is tissue-specific (39). Indeed, *KRAS* mutations are usually detected in colorectal, pancreatic, biliary tract, and lung carcinomas, *NRAS* mutation in malignant melanomas, and *HRAS* mutation in head and neck carcinomas (41, 42). This feature has been investigated in an *adenomatous polyposis coli (APC)*-deficient mouse model where mutations of *KRAS* were able to promote the development of colorectal cancers, while *NRAS* mutations were ineffective (43).

About 40% of colorectal cancers are *KRAS* mutated, 5% NRAS mutated, and rarely *HRAS* mutated. Of note, mutations in different RAS isoforms seems to be mutually exclusive. For this reason, from now on we focus mostly on *KRAS*. *KRAS* mutations are considered to play a pivotal role both in the early phases of malignant transformation of colorectal cells and in the advanced metastatic disease (44). In colorectal cancer, most KRAS mutations are in the codons 12 (about 80%) and 13 (about 15%) of exon 2 and in the codon 146 of exon 4 (about 4%); the remaining are in the codons 59-61 of exon 3 and in the codon 117 of exon 4 (45). Mutation frequency in each hotspot varies significantly among the diverse cancer types, exactly as it happens for the *RAS*-mutated isoforms (38). This could underlie that also the functional consequences of *RAS* mutation could be divergent in different cancer settings, up to assume paradoxical effects as the induction of cellular senescence as reported by Serrano et al. (46). Moreover, in the same cancer type the effects of a *RAS* mutation could vary depending on the codon involved. Indeed, a proteomic study found that in colorectal cancer cells a *KRAS* mutation in codon 12 leads to the overexpression of doublecortin like kinase 1 (DCLK1) and tyrosine-protein kinase MET, while in codon 13 brings to the overexpression of tight junction protein ZO-2 (47).

## Forms of Cell Migration and Invasion

**Figure 2:**
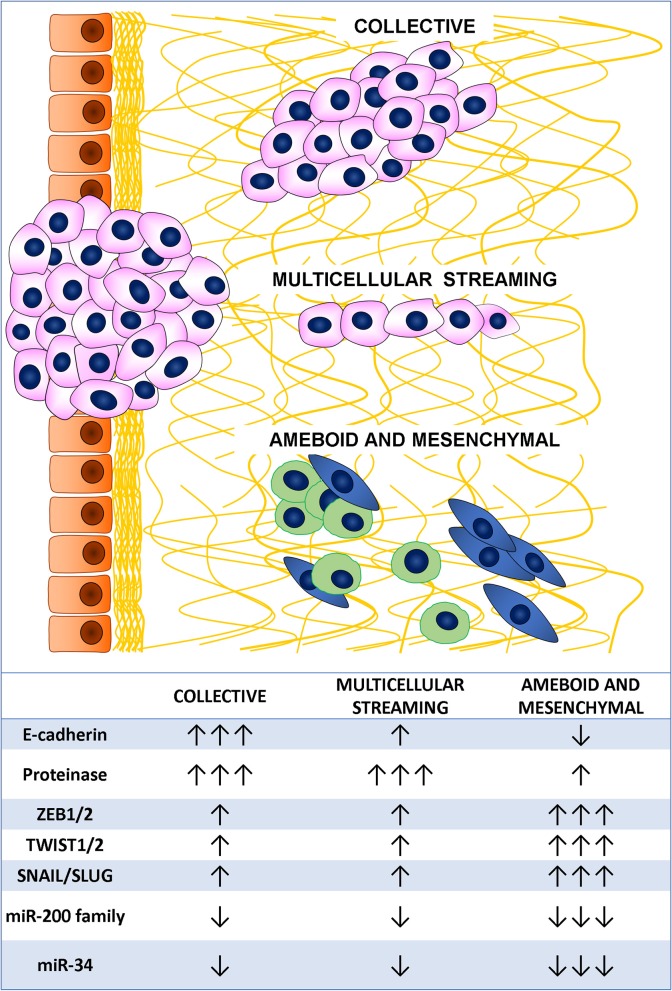
Tumor cell migration mode and main associated markers.

Metastatic dissemination results from tumor cell invasion and migration through the tissues and represents a major challenge in cancer management (48). The cornerstones of these cancer cell characteristics are deregulation of cell-cell adhesion, acquisition of cytoskeletal deformability, gaining of cellular motility, turnover of cell-matrix interactions, and extracellular matrix (ECM) breakdown (49). Cancer invasion and migration are heterogeneous and adaptive processes based on changes in the usual morphology of the cells, generation of new cell polarization, and cell body displacement that finally leads to the translocation of the entire cells. This may happen in different ways (48, 50). Indeed, tumor cell migration may be either individual, with loss of cell-cell junctions, or collective, with retention of intercellular bonds (Figure 2) (49). Two main types of individual cell motility have been recognized: elongated-mesenchymal and rounded-amoeboid modes. As for collective cell migration, it can happen as multicellular streaming or collective invasion. All these patterns of migration are closely linked to the ECM features, resulting from the coordinated actions of actin cytoskeleton, actomyosin contraction, cell polarity, and cell surface receptors interacting with the surrounding cells and ECM structures. Collective and individual invasion may also coexist, enhancing the efficiency of the metastatic process (51).

Individual migration patterns are featured by the absence of tumor cell-cell interactions and are strongly linked to the ECM structure. In the elongated-mesenchymal mode, the high ESM stiffness stimulates the cell to produce actin-rich protrusion, thus the cell assumes a spindle morphology with strong focal adhesion, matrix proteolysis, and actomyosin contractility localized at the rear (52). If the ECM surrounding the tumor is loose, the preferential individual invasion mode is the rounded-amoeboid pattern. The cell in this case forms small, unstable cellular protrusions (blebs or spikes) throughout its surface (53). These result from increased intracellular pressure, low degree of integrin-mediated adhesion, and reduced cell-cell interactions (54). Cyclic expansion and retraction of the cellular protrusion at the leading front of the cell are responsible for the cell progression (55).

In multicellular streaming migration mode, the cells move forming single cell files following the same path and are attracted by chemokines gradients or constrained by the ECM structure (56). Streaming cells can display rounded-amoeboid or spindle-mesenchymal phenotypes and advance by generating traction force on the surrounding ECM with weak and short-lived cell-cell interactions (57).

In collective migration pattern, the tumor advances through the neighboring tissues in compact clusters, strands, or cords of connected cells (49). These patterns are determined by a combination of parameters, such as cellular morphology, cell-cell adhesion, and ECM features. Unlike multicellular streaming migration, the collective migration mode is featured by cohesive cells forming solid strands or cords lined up for two or more cells, even to create broad clusters (58). This pattern is supported by long-lived cell-cell interactions, while the morphology varies according to cell nature, ECM features, and host tissue types (56). Main feature of an invasive multicellular mass is the specialization of the leading edge cells that express a mesenchymal phenotype, generate an integrin-mediated forward traction and ECM rearrangement by enzyme-mediated proteolysis of the surrounding structures (59). Interestingly, this invasion pattern has been described as the slowest migration mode (60), conferring some advantages to the tumor, such as secretion of higher amount of pro-invasive factors and immune escape (61).

## Molecular Regulation of Cellular Plasticity in Colorectal Cancer

The cellular plasticity needed to allow migration of cancer cells is achieved through complex mechanisms finely governed by several genes, most of them encoding for transcription factors. In CRC, the best delineated of these molecular programs driving cellular migration is EMT, that is characterized by the acquisition of a mesenchymal phenotype through tight junction dissolution, disruption of apical-basal polarity, and reorganization of the cytoskeletal architecture (62). A huge amount of studies has shown that EMT plays a pivotal role in cancer progression and metastasis in several tumor types, including CRC (63). EMT requires a precisely regulated cooperation of a complex molecular network, which comprises factors categorized into three groups: the extracellular cues activating EMT (EMT inducers), the transcription factors orchestrating the EMT program (EMT core regulators), and the effector molecules executing the EMT-related cellular transformation (EMT effectors) (64). The best characterized external inducers are the transforming growth factor- β (TGF-β) signaling and the WNT/β-catenin pathway. Both these pathways may induce the expression of the three main family of EMT regulators: (i) the SNAIL family of zinc-finger transcription factors comprising *SNAIL* and *SLUG*; (ii) the zinc finger E-box binding homeobox (ZEB) family of transcription factors including *ZEB1* and *ZEB2*; (iii) the TWIST family of basic helix-loop-helix (bHLH) transcription factors encompassing *TWIST1* and *TWIST2*. The roles of these transcription factors in EMT have been well-established in a variety of cancers including CRC, and most of them showed correlation with the prognosis (65, 66). Final effects of EMT regulators are the overexpression of genes encoding for proteins linked to mesenchymal phenotype, such as vimentin, fibronectin, α-smooth muscle actin, and N-cadherin, and the down-regulation of epithelial markers, such as E-cadherin, claudins, and occludins (64). Post-transcriptional regulation of gene expression by EMT-related miRNAs showed a great impact in promoting epithelial or mesenchymal phenotype targeting specific mRNA (67). Members of the miR-200 family (miR-200a, miR-200b, miR-200c, miR-141, and miR-429) promote epithelial phenotype preventing the translation of *ZEB1* and *ZEB2* mRNA (68–70) that, in turn, act in a negative feedback loop down-regulating the miR-200 family expression (71). Moreover, *ZEB2* is also identified as a direct target of miR-132, miR-192, and miR-335. Downregulation of these miRNAs is usually associated with the acquisition of an aggressive mesenchymal phenotype leading to distant metastasis and dismal prognosis (72, 73). MiR-34a/b/c is another caretaker of the epithelial phenotype through the down-regulation of *SNAIL, SLUG*, and *ZEB1* (74). Suppression of miR-34a/b/c causes up-regulation of SNAIL resulting in the enhanced expression of EMT markers, mesenchymal features, and improved cell invasion and motility.

**Figure 3:**
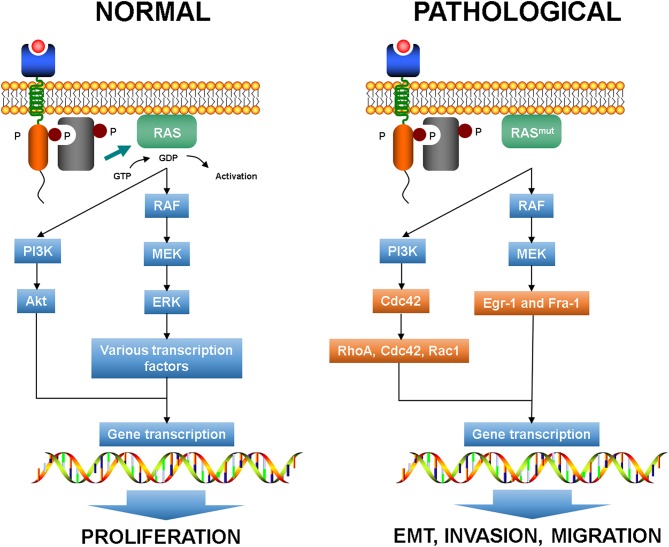
Normal RAS pathways and plasticity-related aberrant pathways. EMT, epithelial to mesenchymal transition.

As above mentioned, *KRAS* mutation is common in CRC and activates several effector pathways involved in cell proliferation, invasion, and migration. In particular, RAS signaling has been reported to play a crucial role in EMT initiation (75, 76). It has been shown that in CRC cell lines mutated KRAS can activate downstream effectors of the PI3K pathway, such as Ras homolog gene family member A (RhoA), Ras-related C3 botulinum toxin substrate 1 (Rac1), and cell division cycle 42 (Cdc42), and in synergy with TGF-β signaling can promote EMT inducing a decrease of E-cadherin expression and an increase of vimentin expression (Figure 3) (40, 77, 78). Thus, it seems that *KRAS* mutation alone is not able to modify the epithelial morphology of CRC cells but requires the cooperation of growth factor cues to accomplish the cell transformation.

RAS activation is a crucial connector between receptor and cytoskeleton during chemotaxis in normal conditions (79). Indeed, PI3K-triggered RAS acts on F-actin forming a coupled excitable system that leads to short-lived RAS-F-actin patches that anticipates the extension of cellular protrusions (80).

Moreover, the activation of MEK1 in the RAS-RAF-MEK cascade allows the enrollment of the downstream effectors Egr-1 and Fra-1 that can promote the expression of SNAIL and SLUG, which in turn downregulate E-cadherin expression (81). In EMT, the pathways that regulate actomyosin and cytoskeleton dynamics drive plasticity and *KRAS* mutation can determine the mode and effectiveness of migration by means RhoA and Rac1 signaling (82, 83).

Several miRNAs were linked to K-RAS-driven tumorigenesis. In experimental models down-regulation of miR-1, Let-7a, miR-16, miR-18a, miR-30a, miR-217, miR-622 results in increased K-RAS expression (84). In particular, miR-30a directly targets KRAS and PI3K inhibiting anchorage-independent growth, cell migration and invasion, and *in vivo* tumorigenesis by KRAS-mutant CRC cells (85, 86). Moreover, low expression of miR-30a has been found in highly metastatic CRC cell lines and liver metastases (86). Clinically, down-regulation of Let-7a was correlated with increased risk of nodal metastasis and with shortened overall and disease-free survival (87).

## Tumor Budding and Mechanisms of Cellular Plasticity in Colorectal Cancer

**Figure 4:**
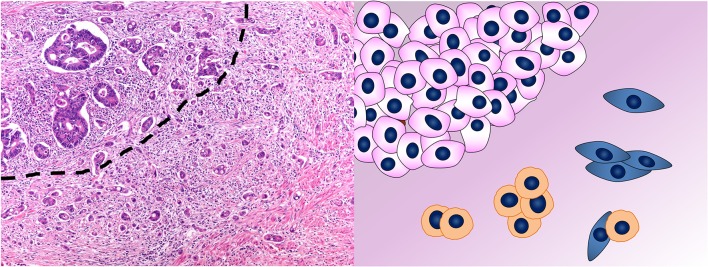
Tumor budding in colorectal cancer. In the photomicrograph, the *dashed line* separates the tumor mass on the top left from the tumor buds on the bottom right. This phenomenon is depicted in the cartoon where single cells or aggregates of up to four cells detach from the mass of neoplastic cells in the top left and infiltrate. Hematoxylin & eosin stain. Original magnification 200x.

According to the definition of the International Tumor Budding Consensus Conference (ITBCC) proposed in 2016 (88) and then validated in 2018 (89, 90), CRC tumor budding (TB) consists of single neoplastic cells or cell clusters of up to four neoplastic cells at the invasive front of the tumor (peritumoral TB) (Figure 4) or within the tumor mass (intratumoral TB) (88). In Western countries, these recommendations were incorporated into the College of American Pathologists (CAP) cancer protocol for patients with primary CRC (91), in the 8th edition of the American Joint Committee on Cancer (AJCC) staging manual (92) and in the European Society for Medical Oncology consensus guidelines (93). This acknowledgment derives from the increasing and established evidences of TB as reliable and independent prognostic factor in CRC, regardless of the scoring method applied for the evaluation (3, 90, 94–96). However, the inclusion of TB in the pathologist report is not yet mandatory, but merely recommended. This is due to its apparent poor reproducibility along with the lack of a standardized scoring system before the ITBCC (97–99). Indeed, TB definition and evaluation method have been controversial throughout its development and different diagnostic criteria are present in the literature (3, 100). The recent agreement reached upon the definition and scoring system method (89) is an essential step to implement TB in the routine CRC assessment.

The morphological feature now called TB was originally described in Japan by Imai in 1949 (101) and firstly reported in the English language literature by Gabbert in 1985 (102). Histologically, TB cells show a more marked atypia than their counterparts in the tumor bulk, thus TB was initially termed “tumor dedifferentiation” (102). Imai, instead, proposed the term “sprouting” to describe the tumor cells detaching from the tumor mass along its invasive edge. Moreover, he suggested to use this feature, peritumoral stromal reaction, and lymphovascular invasion in a prognostic system for gastric cancer (101). Some Japanese researches observed the same phenomenon in CRC (103–105) and it was called TB by Morodomi in 1989 (106). In the last decades, a growing number of data reinforced the value of TB as CRC prognostic marker (107–114). Besides CRC, TB has been found in a variety of other solid tumors, such as oral squamous cell carcinoma (115, 116), invasive ductal breast cancer (117), pancreatic (118), and esophageal cancer (119).

Invasion and metastasis are some of the hallmarks of cancer (120), which requires the ability of tumor cells to detach from the primary tumor, move through the ECM, invade lymphovascular vessels, and finally reach and colonize lymph nodes and distant organs (121, 122). TB is the histological demonstration of this ability, which is intrinsically dynamic. Thus, it is conceivable that tumor buds possess cellular plasticity properties, such as cytoskeletal deformability, motility, and full or partial EMT characteristics (122).

Tumor buds often show typical features of EMT (Table 1): loss of E-cadherin expression, β-catenin translocation in the nucleus (sign of WNT pathway activation), and acquisition of vimentin expression (122). The motile and invasive phenotype of TB cells is depicted by the loss of cell adhesion molecules (such as E-cadherin), overexpression of proteins involved in ECM degradation and cell invasion (such as MMP2, MMP9, and cathepsin B), and cell migration (such as laminin, fascin, and α-smooth muscle actin) (121, 138, 142, 143). However, some studies failed in confirming the expression of the classic EMT-related transcription factors ZEB1, TWIST, SNAIL, and SLUG in tumor buds (131).

**Table 1:** Studies which investigated the *KRAS* status and/or TB in relation to cell morphology and/or cellular plasticity, also considered as EMT, or partial-EMT phenotype.

**References**	**Markers**	**Materials**	**Methods**	**Results**
Alamo et al. (123)	TB*^a^*, CXCR4, 5β- integrin, VEGFA, Serpine-1, and Akt	FFPE from primary CRC and metastasis induced in mice	H&E/IHC/ ELISA	Higher LN metastasis and TB, CXCR4, 5β-integrin, VEGFA, and Serpine-1 overexpression in *KRAS* G12V than KRAS G13D CRC, supporting the higher aggressiveness of CRC harboring this specific mutation
Hammond et al. (47)	DCLK1, proteome, and phosphoproteome	Colon cancer cell lines	–	DCLK1 is amplified and highly overexpressed (mRNA) in *KRAS* G12D cells (transcriptional up-regulation); its amplification is reversed upon suppression of KRAS expression: KRAS has a direct role in regulating DCLK1 expression
Cho et al. (124)	E-cadherin, VIM, RAS, β-catenin	KRAS mutated CRC cell lines/mice	IHC (TMA)/immunoblotting/real time imaging/flow cytometry	KY1022^§^ prevent spindle cell morphology, E-cadherin loss, and VIM over-expression, inhibiting development of metastatic CRC
Centeno et al. (125)	Pan-CK, TB, 50 oncogene, and tumor suppressor genes	FFPE CRC	IHC/NGS	No difference in driver mutations between TB and main tumor (isolated by laser capture microdissection); *KRAS* mutation is not acquired in TBs
De Smedt et al. (126)	Pan-CK, TB, gene expression profile (mRNA), CSM	FFPE CCR	IHC/RNA seq/pathway analysis/clustering	EMT signature (CMS4, mesenchymal phenotype), upregulation of CSC related genes and cellular movement/survival genes, and downregulation of cell growth/proliferation genes in laser microdissected TB compared to tumor bulk, in relation to the CMS taxonomy of CRC
Trinh et al. (90)	TB, CSM	Patient cohorts (AMC-AJCCII-90, LUMC, CAIRO, and CAIRO2) FFPE	H&E/IHC (TMA)	TB is related to CMS4 phenotype (vs. CMS3/2) and with *KRAS* and *BRAF* mutations
Prall et al. (127)	CK18 positive TB, β-catenin, SMAD4, pSTAT3, pERK1/2, KRAS, BRAF [molecular analysis (128)]	FFPE CRC/fresh human CRC tissue for subcutaneous xenografting in T- and B- deficient mice	IHC/ morphometric studies (image J)	In the xenografts TB is reduced, tumor cells are pSTAT3 negative (indicating absence of cytokine/chemokine signaling), some are partially positive for pERK1/2, with maintenance of nuclear β-catenin and SMAD4 immunostainings, and WNT and BMP pathway activation. *KRAS/BRAF* mutational status did not correlate with TB or podia formation in the xenografts
Smit et al. (129)	TrkB, E-cadherin, TWIST, SNAIL, MAPK pathway	Cell culture	Immunoblotting/IF/qRT-PCR/…	TrkB induces an EMT- like transformation in epithelial cells through a Twist-Snail signaling axis, which is dependent on the MAPK pathway. Furthermore, Snail plays a critical and specific role in TrkB-mediated metastasis
Dawson et al. (130)	TrkB, Ki-67, caspase-3, TB	FFPE CRC	IHC (TMA)	Overexpression of TrkB in TB in comparison to main tumor, and association with *KRAS* mutation. High expression of membranous TrkB is an independent adverse prognostic factor. Inverse correlations between Trkb expression and Ki-67 as well as Caspase-3
Yamada et al. (131)	E-cadherin, ZEB1, TWIST, SNAIL, SLUG, TB	FFPE CRC	IHC (TMA)	Absent expression of these EMT markers in TB, but great expression in stromal cells surrounding high grade-TB than in low grade-TB areas
Gibbons et al. (132)	Mir-200 family, ZEB1, ZEB2, CDH1, CDH2, and VIM, (…)	Lung cancer cell lines (3D- culture) derived from mice (*KRAS* and *p53* mutant)	mRNA and miRNA expression profile/qRT-PCR/IF/migration and cytogenetic assay	These tumor cells have a marked plasticity [transit reversibly between epithelial and mesenchymal states, forming highly polarized epithelial spheres in 3D culture that underwent EMT, which is dependent on miR-200 family (decrease during EMT)]. Forced expression of miR-200 abrogated the capacity of these tumor cells to undergo EMT, invade, and metastasize, and conferred transcriptional features of metastasis-incompetent tumor cells. Tumor cell metastasis is regulated by miR-200 expression, which changes in response to contextual extracellular cues
Liu et al. (71)	RAS, miR-200, Rb1, Bmi1, ZEB1, ZEB2, (…)	Cell culture/KRAS mice/NCBI database (GSE11969)	RT-PCR/WB/ISH/H& E/immunostaining/human lung adenocarcinoma microarray analysis	Rb1 pathway status regulates a ZEB1-miR-200 loop downstream of RAS to control expression of Bmi1. Rb1 and ZEB1-miR-200 link RAS to Bmi1 to regulate a cellular choice between oncogene-induced senescence and tumor progression in RAS mutated cells, also triggering EMT
Knudsen et al. (133)	Mir-200b, TB, E-cadherin, β-catenin, and laminin-5γ2	FFPE CRC	IHC/CISH/IF	MiR-200b is downregulated in the TB, but not statistically associated with the expression of the other markers. Loss of membranous E-cadherin and ↑ nuclear β-catenin in the TB (majority of the cases), while laminin-5γ2 expression is upregulated at the invasive front and in the TB (half the cases)
Jang et al. (134)	KRAS, NRAS, BRAF, PIK3CA, TP53, and POLE mutations, and clinicopathological correlations, TB	FFPE CRC	H&E/Sequenom MassARRAY/direct DNA sequencing of KRAS	21 of 34 tumors with high-grade TB had *KRAS mut*; *KRAS* G12D and PIK3CA exon 9 variants were significantly associated with high-grade TB; exons 3 and 4 *KRAS mut* tumors tend to have lymphovascular tumor emboli and perineural invasion
Chang et al. (135)	Clinicopathological features, TB, p16, E-cadherin, β-catenin, HPV-status, KRAS, BRAFV600E	FFPE CRC	H&E/IHC/PCR/HPV-ISH	Comparing early-onset (≤40 years of age) and control (> 40 years) CRC groups, no difference emerged in the occurrence of TB, as well as lymphatic invasion, mucinous histology, or tumor-infiltrating lymphocytes, neither in *KRAS* mutations occurrence
Graham et al. (136)	TB, KRAS, BRAF, MSI, CIMP, clinicopathological features	FFPE CRC	H&E/IHC	High TB (≥10 tumor buds in a 20 × objective field) is present in 32% (179 of 553) of cases, and is associated with advanced pathologic stage, MSI, *KRAS* mutation and on multivariate analysis with a >2 times risk of cancer-specific death
Steinestel et al. (137)	KRAS, BRAF, MMR status, TB, clinicopathological features	FFPE CRC	H&E/IHC/DNA pyrosequencing	TB is associated with infiltrative growth, absence of peritumoral lymphocytic reaction, and blood/lymph vessel infiltration. Neither *KRAS* nor *BRAF* mutations are associated with a certain growth pattern or TB intensity
Zlobec et al. (138)	KRAS, BRAF, MGMT, CIMP, TB	FFPE CRC	H&E/IHC/molecular analysis^*^	TB is not associated with *KRAS, BRAF*, MGMT, or CIMP, but is correlated inversely with MSI-H. TB has an independent role of all these five molecular features and is predicted by MSI status
Pai et al. (139)	TB, BRAFV600E, KRAS, MSI, CIMP	FFPE CRC	H&E/MSI PCR and IHC	In the adenocarcinomas of the proximal colon, no relationship between *KRAS* mutation and TB is identified
Pai et al. (140)	TB, molecular profiling, MSI, clinicopathological features	FFPE surgically resected pT1 CRC (western cohort)	H&E/NGS/MSI PCR and MMR IHC	High grade TB is significantly associated with lymph node metastasis on univariate and multivariate analysis [OR 4.3 (*p* = 0.004)]. No relation with *RAS* mutation is identified
Landau et al. (141)	KRAS, BRAF, MMR status, TB, clinicopathological features	FFPE CRC	H&E/IHC/PCR	Adenocarcinomas of the caecum display the highest frequency of *KRAS* mutations and high TB in the colon (compared to right (non-cecal proximal) and left (distal) adenocarcinomas). Cecal tumor site and high TB are also predictive of poor survival, particularly in stage III/IV of disease

*When the definition of tumor budding differs from up to five cells at the invasive front, the definition applied is reported*.

a *Defined in this study as 10 or fewer cells at the tumor front, counted on IHC (keratin positive cells or clusters) in 3 different tumor fields (400x magnification)*.

§ *KY1022 is a destabilizer of RAS protein and β-catenin*.

* *DNA bisulphite conversion, amplification of modified DNA, and pyrosequencing*.

Tumor buds and their corresponding tumor bulk share the same driver mutations (125). De Smedt et al. found 296 differentially expressed genes by the comparison of neoplastic cells in the tumor mass and those microdissected from the tumor buds (126). TB cells undergo phenotype switching while detaching from the main tumor, with upregulation of genes related to cellular motility and downregulation of genes involved in cell growth and proliferation (126). This is consistent with the hypothesis that migration and proliferation are spatially and temporally exclusive (122). Regarding the CRCSC categories, TB cells showed a gene expression profile consistent with the “mesenchymal phenotype” (CMS4), while the cells in the main tumor had a molecular signature similar to the “canonical subtype” (CMS2) (126). This finding is supported by the results of another study in a large series of CRCs highlighting the association of TB with CMS4 phenotype—a greater number of tumor buds was found in CMS4 than in CMS2 and CMS3 tumors—and *KRAS* mutations (90). A significant association between *KRAS* mutations and the presence of high-grade TB has been reported in CRC (Table 1) (122, 131, 138, 142, 143). *In vitro, KRAS* mutations can induce expression of ZEB1, which promotes EMT, invasion, and metastasis (71, 132). Moreover, TB cells in CRC patients show increased expression of ZEB1 and a concomitant reduction of miR-200b and miR-200c, supporting the association between miR-200 family members and EMT (133). Resistance to anoikis, the cell death mechanism that occurs to non-neoplastic cells when detach from ECM, is a prerequisite for TB cells to survive during invasion. Neurothropic tyrosine receptor kinase B (TrkB) is a potent anoikis suppressor, which is overexpressed in tumor buds and in CRC with high-grade tumor budding and *KRAS* mutations (130). Indeed, RAS signaling promotes TrkB-induced EMT, anoikis resistance, and metastasis through TWIST and SNAIL (129). Morphologically, treatment of *KRAS* mutated cell lines with a destabilizer of β-catenin and RAS proteins can prevent spindle cell morphology as well as E-cadherin loss and vimentin over-expression (124). A xenograft model of CRC was also studied, but *KRAS* mutational status did not correlate with TB or podia formation (127).

## The Prognostic Relevance of Tumor Budding in Colorectal Cancer

TB can be considered as a snapshot of the dynamic process of invasion and a surrogate morphological marker of EMT. The translation into the clinics of TB, for a long time believed as a sign of biological aggressiveness, fits with its demonstration as an adverse and independent prognostic marker in all stages of CRC (Table 2) (3, 90, 94–96, 122, 136, 141, 146, 148). Regardless of the assessing method, evidences suggest that TB has a prognostic effect independent of age, sex, and stage of disease (3, 90, 94–96, 122, 123, 135, 146, 148). TB is usually associated with high tumor grade, advanced stage, lymphovascular invasion, nodal and distant metastasis, locoregional and distant recurrence, and worse overall, disease free, and recurrence free survival (122). The clinical implications are not only prognostic but also therapeutic. In metastatic patients, the presence of high tumor budding can predict resistance to anti-EGFR therapies (149). Moreover, *KRAS* status assessment seems to be useful to identify possible non-responder patients in the metastatic setting (149). Recently, it has been demonstrated that intratumoral TB is related to nodal and distant metastasis in CRC (90, 150–152). Apparently, intratumoral TB has a prognostic effect assessed on a continuous scale (90), and similarly to peritumoral TB has been associated with higher stages, vascular invasion, infiltrative margin, poor survival, and to peritumoral TB itself (122, 147). To date, the prognostic impact of TB has been associated with three major clinical scenarios.

**Table 2:** Selected studies and reviews (^∧^) which investigated tumor budding as a prognostic marker in colorectal cancer.

**References**	**Stage**	**ITB and/or PTB**	**Prognostic parameters associated with TB**
Beaton et al. (95)^∧^	Early CRC	n.a.	A total of 4510 patients from 23 cohort: TB is significantly associated with LN metastasis
Pai et al. (140)	pT1	PTB	High TB is significantly associated with LN metastasis on univariate and multivariate analysis
Cappellesso et al. (3)^∧^	pT1	n.a.	A total of 10,137 patients from 41 studies (heterogeneous TB definition): strong association between the presence of TB and risk of nodal metastasis in pT1 CRC
Okuyama et al. (109, 144)^*^	II vs. III	PTB	TB-positive CRC have worse outcome and more frequently LVI and LN metastasis than TB-negative CRC. TB-positive stage II CRC have similar outcome as TB-negative stage III. TB is an independent prognostic factor in stage II and III CRC (multivariate analysis)
Nakamura et al. (111)	II vs. III	PTB	Significant correlation of TB and LN and distant metastasis, and survival. Similar survival rates between high TB stage II tumors and stage III disease
Wang et al. (145)	T3N0M0	PTB^#^	High-TB is associated with infiltrative growth pattern and LVI. 5-year cancer-specific survival is poorer in high vs. low TB. TB is an independently prognostic (multivariate analysis)
Petrelli et al. (96)^∧^	II	n.a.	A total of 1,652 patients from 12 studies (heterogeneous TB definition): TB is associated with worse 5-y OS in stage II CRC, in particular in pT3N0M0 patients. High-grade TB is associated with an increased risk of death
Zlobec et al. (138)	I–IV	PTB	High grade TB is an independent prognostic factor even in presence of genetic and epigenetic aberrations (those investigated in this study). TB is predicted by MSI status
Steinestel et al. (137)	I–IV	PTB	TB is significantly associated with infiltrative growth, absence of peritumoral lymphocytic reaction, and blood and lymph vessel infiltration
Graham et al. (136)	I–IV	PTB	TB is associated with LVI, metastasis, MSI, *KRAS* mutation, 5-y survival. High TB is associated with 2.5 times increased risk for cancer-related death compared to no TB. More than 10 budding cells/ × 200 field is a good cut-off for high TB
Rogers et al. (94)^∧^	I-IV	n.a.	A total of 7,821 patients from 34 papers (heterogeneous TB definition): TB in CRC is strongly predictive of lymph node metastases, recurrence, and cancer-related death at 5 years
Jang et al. (134)	I–IV	PTB	High-grade TB is significantly associated with conventional histological G, T, N, and M stages, LVI, infiltrative growth pattern, and *KRAS* mutations; patients with low-grade TB had high 4-years DFS and DSS rates, compared to those with high-grade TB
Landau et al. (141)	I–IV	PTB	Adenocarcinomas of the caecum display the highest frequency of *KRAS* mutations and high TB in the colon (compared to right [non-cecal] and left [distal] adenocarcinomas). High TB and cecal tumor site are predictive of poor survival, particularly in stage III/IV of disease
Oh et al. (146)	I-III	PTB	High TB is associated with adverse histologic features such as elevated levels of preoperative carcinoembryonic antigen, advanced stage, poor histology, and the presence of LVI/perineural invasion. High budding is an independent poor prognostic factor in DFS and OS, whereas tumor-budding positivity itself was not an independent prognostic factor (multivariate analysis)
Lugli et al. (147)	I–IV	ITB and PTB	ITB correlates with PTB and is independently associated with a shorter survival time. In MMR-proficient tumors: high-grade ITB is associated with right-sided location, advanced T and N stage, LVI, infiltrating tumor margin, and shorter survival time; MMR–deficient cancers: high ITB is linked to higher tumor G, vascular invasion, infiltrating tumor margin, and more unfavorable survival time
Trinh et al. (90)	I–IV	ITB and PTB	Adverse prognostic factor independent of age, stage, and sex. Independent in metastatic setting and in mixed stage cohort

When the definition of tumor budding differs from up to five cells, the paper is highlighted (*).

# *Usually but not always at the invasive front*.

*CMS, consensus molecular subtype; CRC, colorectal cancer; ITB, intratumoral budding; LN, lymph node; LVI, lymphovascular invasion; MMR, mismatch repair protein; MSI, microsatellite instability; n.a., non-applicable, PTB, peritumoral budding; TB, tumor budding*.

First, in CRC infiltrating the submucosa (categorized as pT1 according to the current staging system), TB is an accurate predictor of nodal metastasis (3, 88, 95, 153–155). A recent meta-analysis including over a thousand of patients with endoscopically removed pT1 CRCs has shown that tumors with TB are strongly associated with lymph node involvement (3). In a western cohort of 116 surgically resected pT1 CRCs, high grade TB has been significantly associated with lymph node metastasis on univariate and multivariate analysis (140). While the Japanese Society for Cancer of the Colo-Rectum has already incorporated TB among the mandatory prognostic variables for pT1 CRC reports, in Western countries this has not yet happened. However, the available evidences strongly support its incorporation also in Western guidelines to improve lymphadenectomy planning (3, 88, 89, 140).

Second, in stage II CRC (namely a tumor without nodal and distant metastasis) the presence of high-grade TB confers a more aggressive behavior similarly to stage III CRC (namely a tumor with nodal metastasis but without distant metastasis) (96, 109, 111, 113, 126, 144, 145, 156). A metanalysis including over a thousand and a half stage II CRC patients highlighted that tumors with high grade TB are associated with worse overall survival, with a difference of survival of about 25%, mostly in pT3N0M0 patients (96). The survival rate of stage II CRC patients stratified as low or high grade TB vs. stage III CRC ones has been directly studied showing significantly differences depending on TB level (111). In particular, the survival rates of stage II CRC patients with high grade TB resulted comparable to those of patients with metastatic disease. These findings raise the opportunity of offering adjuvant chemotherapy to these patients, since they are expected to have a more aggressive disease.

Third, pre-operative biopsies could benefit of intratumoral TB assessment. Indeed, in CRC surgical samples intratumoral and peritumoral TB are strongly related and associated with a shorter survival (147). Moreover, high-grade intratumoral TB correlates with higher tumor grade, more advanced primary tumor, lymphatic and vascular invasion, and nodal metastasis (147). Intratumoral TB could be used as predictive parameter in the selection of candidates for neo-adjuvant therapy (88, 90).

## Conclusion Remarks

The deepening of the knowledge on the molecular mechanisms linking common gene mutations, such as those affecting *RAS*, to specific gene-expression profiles, tumor cell characteristics, and biological behavior will disclose novel opportunities for the prevention, detection, and tailored treatment of CRC.

## Author Contributions

VM and LN reviewed the literature, wrote the first draft, and edited the manuscript. RC planned the article, coordinated and supervised the work, reviewed the literature, wrote the first draft, and edited the manuscript.

### Conflict of Interest

The authors declare that the research was conducted in the absence of any commercial or financial relationships that could be construed as a potential conflict of interest.

## Abbreviations

CIN: chromosomal instability
CSS: chromosomal stability
CIMP-N/L/H: CpG island methylator phenotype-negative/low/high
MSS: microsatellite stability
MSI-L/H: microsatellite instability-low/high
EMT: epithelial to mesenchymal transition
ECM: extra-cellular matrix
TB: tumor budding
APC: adenomatous polyposis coli
KRAS: Kirsten rat sarcoma viral oncogene homolog
NRAS: neuroblastoma RAS viral oncogene homolog
HRAS: Harvey rat sarcoma viral oncogene homolog
TP53: tumor protein p53
TGF-β: transforming growth factor β
FAP: familial adenomatous polyposis
MUTYH: mutY DNA glycosylase
MAP: MUTYH-associated polyposis
CRCSC: CRC Subtyping Consortium
CMS: consensus molecular subtypes
GDP: guanosine diphosphate
GTP: guanosine-5′-triphosphate
RASGEF: recruit guanine nucleotide exchange factor
PI3K: phosphoinositide 3-kinase
MEK: mitogen-activated protein kinase kinase
ERK: extracellular signal-regulated kinase
DCLK1: doublecortin like kinase 1
ZEB: zinc finger E-box binding homeobox
TrkB: tyrosine receptor kinase B
EGFR: epidermal growth factor receptor.
